# Is the process of delivery of an individually tailored lifestyle intervention associated with improvements in LDL cholesterol and multiple lifestyle behaviours in people with Familial Hypercholesterolemia?

**DOI:** 10.1186/1471-2458-12-348

**Published:** 2012-05-14

**Authors:** Karen Broekhuizen, Judith Jelsma GM, Mireille van Poppel NM, Lando Koppes LJ, Johannes Brug, Willem van Mechelen

**Affiliations:** 1 Department of Public and Occupational Health, EMGO+ Institute for Health and Care Research, VU University Medical Centre, Amsterdam, the Netherlands; 2 TNO, Division Work and Employment, Hoofddorp, The Netherlands; 3 Department of Epidemiology and Biostatistics, EMGO+ Institute for Health and Care Research, VU University Medical Centre, Amsterdam, the Netherlands

**Keywords:** Counselling, Computer tailoring, Cholesterol, Lifestyle behaviours, Dose, Fidelity, Motivational interviewing, Process evaluation, Efficacy

## Abstract

**Background:**

More insight in the association between reach, dose and fidelity of intervention components and effects is needed. In the current study, we aimed to evaluate reach, dose and fidelity of an individually tailored lifestyle intervention in people with Familial Hypercholesterolemia (FH) and the association between intervention dose and changes in LDL-Cholesterol (LDL-C), and multiple lifestyle behaviours at 12-months follow-up.

**Methods:**

Participants (n = 181) randomly allocated to the intervention group received the PRO-FIT intervention consisting of computer-tailored lifestyle advice (PRO-FIT*advice) and counselling (face-to-face and telephone booster calls) using Motivational Interviewing (MI). According to a process evaluation plan, intervention reach, dose delivered and received, and MI fidelity were assessed using the recruitment database, website/counselling logs and the Motivational Interviewing Treatment Integrity (MITI 3.1.1.) code. Regression analyses were conducted to explore differences between participant and non-participant characteristics, and the association between intervention dose and change in LDL-C, and multiple lifestyle behaviours.

**Results:**

A 34% (n = 181) representative proportion of the intended intervention group was reached during the recruitment phase; participants did not differ from non-participants (n = 623) on age, gender and LDL-C levels. Of the participants, 95% received a PRO-FIT*advice log on account, of which 49% actually logged on and completed at least one advice module. Nearly all participants received a face-to-face counselling session and on average, 4.2 telephone booster calls were delivered. None of the face-to-face sessions were implemented according to MI guidelines. Overall, weak non-significant positive associations were found between intervention dose and LDL-C and lifestyle behaviours.

**Conclusions:**

Implementation of the PRO-FIT intervention in practice appears feasible, particularly PRO-FIT*advice, since it can be relative easily implemented with a high dose delivered. However, only less than half of the intervention group received the complete intervention-package as intended. Strategies to let participants optimally engage in using web-based computer-tailored interventions like PRO-FIT*advice are needed. Further, more emphasis should be put on more extensive MI training and monitoring/supervision.

**Trial registration:**

NTR1899 at ww.trialregister.nl.

## Background

In public health research, much emphasis is put on the evaluation of interventions in randomised controlled trials (RCTs). Conducting a process evaluation is indispensable, since it helps to explore if the intervention was adopted and implemented as planned, and how and why the intervention worked or not [1-4]. Public health interventions often are complex interventions combining different potential active ingredients tailored and targeted to context. Complex interventions often prove efficacious in RCTs conducted in well-controlled circumstances, but less effective in practice [5,6].

In 2009, we started the PRO-FIT project (PROmoting a healthy lifestyle in people with Familial Hypercholesterolemia (FH) through an Individually Tailored lifestyle intervention) [7]. The purpose of the PRO-FIT project was to reduce cardiovascular disease (CVD) risk by promoting a healthy lifestyle in people with FH. The intervention aimed to reduce CVD risk by improving awareness of CVD risk, by improving one’s motivation to obtain and maintain a healthier lifestyle (regarding physical activity, saturated fat intake, fruit intake, vegetables intake, smoking and compliance to statin therapy). Basically, the intervention was a combination of two components: I) computer-tailored lifestyle advice (called: PRO-FIT*advice), and II) counselling (face-to-face and telephone booster calls) using Motivational Interviewing (MI).

In the past years, both computer-tailored lifestyle advice and MI-guided counselling have been tested in RCTs for effects on changes in separate health behaviours. Print-delivered as well as on-line computer-tailored health advice has been shown to be efficacious in changing behaviours, even though effect sizes mostly are small [8-12]. Advantages of using the internet as the channel for tailored health advice is the opportunity to provide interactive, individualised interventions to large numbers of people that match each person’s unique characteristics, circumstances, beliefs, motivation to change and behaviour [13-15]. Despite the evidence for efficacy of these interventions, earlier efficacy studies have indicated that the use of and exposure to the content of internet interventions may often not be optimal [16,17]. Especially for people of lower socio-economic positions [18] and older age [19], it may be less likely to save and re-read interactively delivered feedback, due to difficulties to read or process information from a computer screen. Apparently, once delivered, affecting the received dose and further use of the intervention is challenging. Knowledge about delivery, use and efficacy could help us to gain insight in efficacious components of web-based interventions. Counselling according to MI has been regarded as a potentially promising tool to encourage health behaviour change [20-22]. MI has been defined as a ‘client-centred, directive method for enhancing intrinsic motivation to behaviour change by exploring and resolving ambivalence’ [23]. The therapeutic relationship is a partnership with respect of client autonomy and relies upon identifying and mobilising the client’s intrinsic values and goals to stimulate behaviour change [21]. However, the impact of MI largely depends on the fidelity of intervention delivery [24,25]. Clearly, more insight in the association between reach, dose and fidelity of intervention components and efficacy is needed.

**Figure 1:**
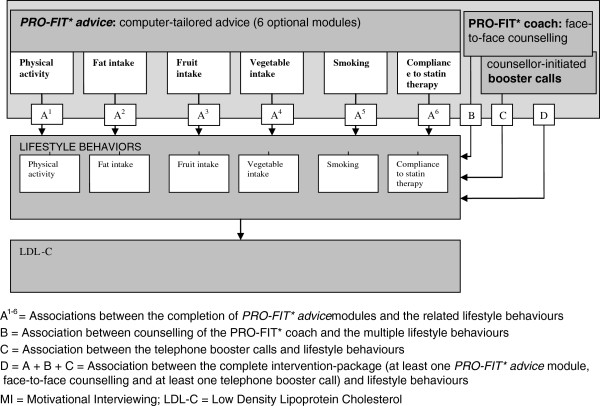
**The PRO-FIT intervention and assumed efficacy pathways.** Legend: This figure shows a schematic overview of the PRO-FIT intervention, including the assumed efficacy pathways of the intervention (associations A-D). It was assumed that the dose of: A) PRO-FIT*advice, B) face-to-face counselling, C) telephone booster calls, and D) the complete intervention-package, was positively associated with change in lifestyle behaviour and LDL-C levels.

Earlier, we investigated the efficacy of the PRO-FIT intervention on multiple lifestyle behaviours (smoking, physical activity, fruit intake, vegetable intake, and compliance to statin therapy) [26] and on LDL-Cholesterol (LDL-C) [27]. The aim of the present paper is twofold: first to evaluate the reach, dose (delivered and received) and fidelity of the PRO-FIT intervention, and second to investigate whether the dose of: A) PRO-FIT*advice, B) face-to-face counselling, C) telephone booster calls, and D) the complete intervention-package, was associated with change in lifestyle behaviour and LDL-C levels (further called: associations A-D) (Figure 1).

## Methods

### The PRO-FIT intervention

Participants of the PRO-FIT trial were recruited from the national cascade screening program of the Foundation for the Identification of Persons with Inherited Hypercholesterolemia (StOEH). Within this program, the StOEH actively approaches first and second degree relatives of index patients (that is, clinically diagnosed FH patients with a known mutation) about their potential risk by mail. A genetic field worker telephones and, if the family member agrees to participate, makes an appointment for testing at home. If the results of DNA analysis are positive, first and second degree relatives are approached and offered testing, and so on. No further counseling is given within the screening program.[28] Within the PRO-FIT project, individuals were invited who were diagnosed with FH by StOEH from January 1^st^ 2007 to April 15^th^ 2009, no longer than 2 years before the start of the project. Access to internet, sufficient fluency in Dutch, residency < 150 km radius from Amsterdam, age 18–70 and LDL-C > 75^th^ percentile were eligibility criteria. People were invited by postal mail and telephoned in case of no response. When people decided to participate, the study procedure was explained by telephone. After randomised allocation to the intervention group, participants were encouraged to visit a weblink referring to the project website, on which they could log on to a personal PRO-FIT*advice account. This account gave access to six tailored advice modules on smoking, physical activity, saturated fat intake, fruit intake, vegetables intake and compliance to statin therapy. Each module required the completion of a screening questionnaire. Subsequently, on-screen personalised feedback was tailored to personal performance level (current lifestyle behaviour), awareness of one’s own performance, as well as personal motivation to change, outcome expectations, attitude and self-efficacy. Personalised feedback to compliance to statin therapy was tailored to knowledge and personal beliefs about (the effect of) statin therapy, potential side effects of the prescribed drug and current compliance. After finishing a module, participants were encouraged to make action plans to change behaviour (except for the advice module on compliance to statin therapy). Thereafter, in a face-to-face session, the participant and the personal coach together further established the level of the participant’s knowledge/awareness about FH and cardiovascular risk factors, according to the assessment(s) and advice(s) within the participant’s personal PRO-FIT*advice account. Ambivalence and barriers related to the recommended behaviour changes were explored in a face-to-face session based on MI techniques. Further, the participant was encouraged to plan five additional counsellor-initiated booster telephone calls, according to their need for additional counselling, intended to support the participant’s behavioural changes and to provide further brief MI to encourage the planned behavioural changes. The two personal coaches had lifestyle coaching and nursing/ teaching backgrounds and had received an additional 3-day MI workshop, incorporating both introductive lessons and practical training sessions with professional actors. A schematic overview of the intervention can be found in Figure 1, including the assumed efficacy pathways of the intervention (associations A-D). A more detailed description of the PRO-FIT intervention can be found elsewhere [7]. The ethical principles of the Helsinki Declaration were followed and the PRO-FIT project was approved by the Medical Ethics Committee of the VU University Medical Centre (reference number: NL23932.029.08). All participants gave written informed consent.

### Theoretical framework

The RE-AIM evaluation framework conceptualised the evaluation of the translatability of an intervention and included reach, efficacy, adoption, implementation and maintenance [29]. Linnan and Steckler also included implementation among their key components of a process evaluation including context, reach, dose delivered, dose received, fidelity, implementation and recruitment [3]. Both authors agree on reach, implementation, including dose and fidelity as important factors for process evaluations. Generally, reach is defined as the number of people of the target population taking part in the project and their representativeness with regard to the target population. Dose is either defined as ‘dose delivered’, i.e. the number of components of the intervention delivered, or as ‘dose received’, i.e. the extent to which the participants used the components of the intervention as intended. Fidelity is defined as the extent to which the intervention was implemented as intended.

Guided by Saunders and colleagues, a process evaluation plan was developed in order to monitor and document the implementation of an intervention [30]. In this plan, the evaluated intervention was described in detail, including its specific strategies as well as what would be entailed in a complete and acceptable delivery of the intervention. Consequently, a list of potential process evaluation questions and measures was made and answered by using self-formulated methods (see Table 1).

**Table 1:** **Process evaluation plan formulated according to Saunders et al[**[30]**]**

**Process evaluation question**	**Complete and acceptable delivery**	**Process measure**
How many people of the target population took part in the project? How representative is the intervention group for the study population? **(Reach)**	The intervention group is comparable to the study population.	Self-report, StOEH client database
To how many participants was a PRO-FIT*advice account provided? **(Dose delivered)**	A log on account was provided to all (100%) participants.	Coach logs/project database
To what extent did participants actively engage in using PRO-FIT*advice as intended, with regard to logging on, the number of modules finished and action planning? **(Dose received)**	All participants (100%) logged on and completed at least one of the modules of PRO-FIT*advice. Action planning was optional.	Website use data
How many participants received a visit from a personal lifestyle coach? **(Dose delivered)**	All (100%) participants received a visit from the lifestyle coach.	Coach logs/project database
To what extent was face-to-face counselling delivered as planned by MI guidelines? **(Fidelity)**	All (100%) face-to-face counselling sessions were delivered according to MI guidelines.	The Motivational Interviewing Treatment Integrity (MITI 3.1.1.) code
How many telephone booster sessions were provided? **(Dose delivered)**	1-5 telephone booster sessions were delivered.	Coach logs

### Reach

In order to assess the number of people with FH that took part in the project, as well as how representative the participants in the intervention group were for the study population and non-participants (people who did not respond to the invitation to participate, or people who chose not to participate), the StOEH client database, as well as the PRO-FIT client database were consulted. Differences between participants and non-participants in main characteristics (age, gender, and LDL-C levels) were explored.

### Dose

The number of participants who had received a PRO-FIT*advice log on account, a face-to-face counselling session and subsequent telephone booster calls (dose delivered), was assessed by logs that were kept by the coaches and stored in the project database. We aimed at a 100% delivery of the intervention components and delivery of one to five telephone booster calls. The way participants used the PRO-FIT*advice log on account (dose received) was assessed by exploring participants’ log on behaviour (% of participants that logged on), as well as participants’ actions on the PRO-FIT* advice account (number of modules finished,% of participants that had made online action plans) by means of log on rates and website use data.

### Fidelity

Whether face-to-face counselling sessions were implemented as planned according to MI guidelines (i.e. MI fidelity) was assessed by two MI experts, following the Motivational Interviewing Treatment Integrity code (MITI 3.1.1.) [31]. The MI experts were attached to the Foundation Centre for Motivation and Change (Hilversum, the Netherlands), that works in cooperation with the International Motivational Interviewing Network of Trainers (Virginia, US; http://www.motivationalinterviewing.org), and were trained in coding fidelity using the MITI 3.1.1. For this assessment, a random sample of 20 audio taped counselling sessions (10 sessions of each lifestyle coach; approximately 10% of all sessions) was drawn. A verbatim transcript [32] of each drawn session was evaluated and resulted in two scores: a global score and behaviour counts. The global score captured an overall impression of the conversation on a 5-point Likert scale for the following 5 dimensions: Evocation, Collaboration, Autonomy/Support, Direction and Empathy. In addition, the behaviour counts capture specific behaviours of the lifestyle coach, such as the number of open/closed questions, simple/complex reflections, MI (non)adherent utterances and provision of information. We aimed for 100% of the counselling sessions to be provided according to MI. Counselling sessions were considered MI if the following conditions were met: average of global scores ≥ 3.5, reflection to question ratio is in favour of reflection, >50% open questions, >40% complex reflections and >90% MI-adherent utterances. The total scores were weighed for the number of counselling sessions conducted by each coach.

### Change in lifestyle behaviours

The level of physical activity was measured by the Short QUestionnaire to ASsess Health-enhancing physical activity (SQUASH) and was expressed as minutes of moderate to vigorous physical activity performed per week [33]. Saturated fat, fruit and vegetables intake were measured by the short Dutch questionnaire on total and saturated fat intake and on fruit and vegetable intake. From this questionnaire, a score for saturated fat intake, ranging from 0 (lowest) to 80 (highest) fat points was computed, as well as servings of fruit and grams of vegetables per day [34-36]. Smoking behaviour was assessed by a self-reported measure, resulting in a score of 0 (non-smoker) or 1 (smoker) [37]. The five-item Medication Adherence Report Scale (MARS-5) was used to measure self-reported compliance to statin therapy. Scores on five items were combined to a total score ranging from 5 (lowest) to 25 (highest). Participants with a score of 25 were categorised as compliant to statin therapy, others (score < 25) as non-compliant [38].

### Change in LDL-C

At baseline and 12-month follow-up, the participants’ LDL-C was assessed at the participant’s home with fasting finger stick samples analysed on a Cholestech LDX desktop analyser (Cholestech, Hayward, USA). This portable analyser is capable of providing a lipid profile in approximately 5 minutes. The reproducibility and precision of lipids measurement using the LDX analyser are within the guidelines of the National Cholesterol Education Program (NCEP) [39,40]. The Cholestech LDX analyser has been validated for point-of-care lipid measurements in clinical practice [41].

### Statistical analyses

Differences in age, gender, and LDL-C levels between participant and non-participant characteristics were checked with linear and logistic regression analyses for each variable separately. Associations between intervention dose and lifestyle behaviours and LDL-C (associations A-D) were explored with linear (for physical activity, fat/fruit and vegetables intake and LDL-C levels) and logistic (for smoking and compliance to statin therapy) regression analysis with the following independent variables: logged on at PRO-FIT*advice and advice module completed (yes/no) (association A), face-to-face counselling received (yes/no) (association B), number of telephone booster calls (association C), and the complete intervention-package (at least one PRO-FIT*advice module, face-to-face counselling and at least one telephone booster call) received (yes/no) (association D). The post-test scores of the dependent variables were regressed to the baseline measures. Effect parameters (regression coefficient (beta) or odd’s ratio (OR)) either indicated a positive association if LDL-C/lifestyle behaviours improved when regressed to the intervention dose, or a negative association if vice versa. An association was considered as significant if p < 0.05.

## Results

### Reach

During the six months of recruitment for the PRO-FIT project, nearly 6200 people in the Netherlands were screened by StOEH, of whom an averaged 35% actually did have FH [42]. Invitation brochures were send to 986 people who were screened by StOEH and who were positively diagnosed with FH. Of those, 340 (34%) responded and agreed to participate. This number included 23 family members of invited people who spontaneously responded and met the eligibility criteria. Reasons for not participating were mainly a lack of interest and time, and reporting to ‘already have a healthy lifestyle’. The participants did not differ from the non-participants (those who did not respond to the invitation and those who refused to participate; N = 623) in age (beta:0.23; 95% CI:-1.85-2.31) and gender (OR:0.89; 95% CI:0.68-1.16), but did with regard to LDL-C levels (beta:-0.35; 95% CI:-0.63- - 0.07) (see Table 1). The majority (57%) of the study sample was female, middle-aged (mean age = 45.3 years) , and had elevated (≥2.5 mmol/l) LDL-C levels. No significant baseline differences between intervention and control group were found. During the PRO-FIT project, five participants in the intervention group dropped out (i.e. their participation was discontinued with a given reason). Their reasons for discontinuation were no motivation (n = 1), no interest (n = 2), death (n = 1), and health constraints (n = 1).

### Dose

An account to use the online PRO-FIT*advice, was provided to 172 (95%) of the 181 participants in the intervention group (see Table 2). The remaining 5% (9 participants) explicitly reported to have no interest in using PRO-FIT*advice and therefore, received no log on information. Subsequently, nearly all participants (99%) in the intervention group were visited by the lifestyle coach. Furthermore, on average of 4.2 telephone booster calls per respondent were conducted. The main reasons for not receiving subsequent booster calls was no perceived need for additional counselling because respondents regarded the lifestyle as healthy.

**Table 2:** Baseline characteristics of responders and non-responders and dose of the PRO-FIT intervention in the intervention group

	**Intervention group**	**Control group**	**Non-responders**
Gender (% female; N)	57.1; N = 181	56.3; N = 159	53.8; N = 623
Age (years, mean ± SD; N)	44.7 (12.9); N = 181	45.9 (13.0);N = 159	45.1 (15.8); N = 623
LDL-C (mmol/l, mean ± SD; N )	3.7 (1.3); N = 1463	3.7 (1.2); N = 130	4.05 (1.33); N = 110
Participants that received a PRO-FIT*advice log on account	95% (172/181)		
Participants that logged on at PRO-FIT*advice and completed at least one module	49% (85/172)		
Participants that logged on at *PRO-*			
FIT*advice and completed the module on:			
Physical activity	41% (71/172)		
Fat intake	35% (60/172)		
Fruit intake	37% (64/172)		
Vegetable intake	34% (59/172)		
Smoking	14% (24/172)		
Compliance to statin therapy	26% (44/172)		
Participants that formulated an action plan at PRO-FIT*advice for at least 1 of the modules^1^	31% (53/172)		
Participants that received face-to-face counselling	99% (179/181)		
Telephone booster calls delivered (mean ± SD; N)	4.2 (1.3); N = 181		
Participants that logged on, finished at least 1 module, received face-to-face counselling and at least 1 telephone booster call (=complete intervention-package)	47% (85/181)		

N=sample size; SD = standard deviation; Significant differences in baseline characteristics between control and intervention group (P < 0.05) are printed in bold font.

^1^ Action planning was not possible in the advice module on compliance to statin therapy.

Of the 172 participants in the intervention group who had received a log on account, 85 (49%) actually logged on to PRO-FIT*advice, and completed at least one of the six advice modules. The most popular module, based on completion rates, was physical activity (41%), followed by fruit intake (37%), fat intake (35%), vegetable intake (34%), smoking (14%) and compliance to statin therapy (26%). Nearly one third (31%) completed at least one module and made an action plan online. Although revisiting the website was not so explicitly encouraged, 7% did. The complete intervention-package as intended, requiring log on at PRO-FIT*advice, the completion of at least one module, face-to-face counselling and at least one received telephone booster call, was delivered to 47% of the intervention group.

The five drop-outs all received a log on account to PRO-FIT*advice and two of them logged on. Consequently, they all received face-to-face counselling and an average of 2 telephone booster calls.

### Fidelity

Eighty-five percent of the face-to-face counselling sessions were performed by coach 1, and 15% by coach 2. In Table 3, the extent to which MI was applied during the face-to-face counselling sessions by the two coaches is shown. The global scores and behavioural counts indicate that none of the sessions was implemented according to MI guidelines. Significant differences in counselling performance between the two coaches were found for using (complex) reflections, the number of MI adherent statements, the reflection to question ratio, directiveness and showing empathy.

**Table 3:** MI fidelity within a sample of face-to-face counselling sessions (n = 20) according to the MITI scoring instrument

	**Global scores^1^ (recommended)**			**Behaviour counts^2^ (recommended)**			
	**(mean (SD))**						
	**Empathy**	**Spirit**	**Direction**	**RF:QU**	**OQ (%)**	**CR (%)**	**MIA (%)**
	**(>3.5)**	**(>3.5)**	**(>3.5)**	**(in favour of RF) (mean (SD))**	**(>50%)**	**(>40%)**	**(>90%)**
Coach 1	3.1 (0.9)	2.7 (1.0)	3.4 (0.7)	1.09 (0.35)	21 (12)	42 (21)	87 (9)
Coach 2	1.5 (0.7)	2.2 (0.9)	2.6 (1.1)	0.68 (0.30)	19 (13)	23 (14)	62 (17)
Total^3^	2.9	2.7	3.3	1.03	21	39	83
(100%)							

^1^ The global scores capture an overall impression of the conversation on a 5-point Likert scale for the following 5 dimensions: empathy, spirit (evocation, collaboration and autonomy) and direction.

^2^ Behaviour counts incorporate: RF:QU=ratio reflections to questions; OQ=percentage open questions; CR=percentage complex reflections; MIA=percentage motivational interviewing adherent; Spirit=combination of evocation, collaboration and autonomy.

^3^ Aggregated scores weighted for the number of counselling sessions conducted by each coach (coach 1: 85%, coach 2: 15%)

Significant differences (p < 0.05) in scores between coaches are printed in bold.

### Associations between intervention dose and change in lifestyle behaviours and LDL-C levels

#### Association A

The association between the dose of each PRO-FIT*advice module (A^*1-6*^*) and change of the related lifestyle behaviour and LDL-C.*

As was assumed in Figure 1, there were positive associations between the completion of each advice module and the related behaviour, except for vegetable intake, and logging on and completing at least one advice module was also positively associated with change in LDL-C (see Table 4) However, these associations were not statistically significant.

**Table 4:** Association (regression coefficient beta/odd’s ratio (OR) and 95% confidence interval (CI)) of dose of PRO-FIT*advice and counselling with post-test LDL-C and multiple lifestyle behaviours, adjusted for baseline levels of the dependent variable, in the intervention group (n = 181)

	**LDL-C**	**MVPA**^**1**^	**Fat intake**	**Fruit intake**	**Vegetable intake**	**Smoking**	**Compliance to statin therapy**
	**mmol/l**	**minutes/wk**	**fat points/day**	**servings/day**	**grams/day**	**yes**	**yes**
	**beta**	**beta**	**beta**	**beta**	**beta**	**OR**	**OR**
	**95% CI**	**95% CI**	**95% CI**	**95% CI**	**95% CI**	**95% CI**	**95% CI**
Participants who had logged on at PRO-FIT*advice and completed at least one advice module:	−0.18						
	*−0.45_0.09*						
Participants who had logged on at PRO-FIT*advice and completed the module on:							
Physical activity	−0.09	0.16					
	*−0.37-0.19*	*−0.14-0.45*					
Fat intake	−0.13		−0.51				
	*−0.42-0.16*		*−1.55-0.54*				
Fruit intake	−0.13			0.19			
	*−0.41-0.16*			*−0.05-0.43*			
Vegetable intake	−0.13				−7.13		
	*−0.42-0.15*				*−25.18_10.92*		
Smoking	−0.06					0.11	
	*−0.44-0.32*					*0.01_1.25*	
Compliance to statin therapy	−0.11						1.09
	*−0.42-0.19*						*0.41_2.93*
Participants who had received face-to-face counselling	N/A^2^	N/A^2^	N/A^2^	N/A^2^	N/A^2^	N/A^2^	N/A^2^
Telephone booster calls delivered (mean, SD)	0.06	−0.04	0.26	−0.03	−4.66	1.00	1.02
	*−0.06-0.17*	*−0.10-0.17*	*−0.16-0.68*	*−0.13-0.07*	*−11.94_2.63*	*0.61_1.64*	*0.69_1.51*
Participants who had logged on, finished at least 1 module^3^, received face-to-face counselling and at least 1 telephone booster call (=complete intervention-package)	−0.18	0.10	−0.50	0.16	−6.87	0.11	0.90
	*−0.45-0.09*	*−0.20-0.40*	*−1.56-0.56*	*−0.08-0.40*	*−25.09_11.36*	*0.01_1.25*	*0.33_2.44*

^1^ MVPA=moderate to vigorous physical activity. Due to skewed data, log-linear regression was conducted. Therefore, the beta should be interpreted as follows: a 1% increase of the independent variable is associated with a beta% increase in physical activity.

^2^ Due to minimal variation in dose delivered, no association between dose delivered and efficacy could be tested.

^3^ For LDL-C this means at least one module, for the lifestyle behaviours, this means the related advice module (e.g. for physical activity, the completion of the physical activity module).

Significant associations between dose and efficacy (p < 0.05) are printed in bold. Effect parameters (beta regression coefficient or odd’s ratio (OR)) either indicated a positive association if LDL-C/lifestyle behaviours improved when regressed to the process, or a negative association if vice versa.

#### Association B

The association between the dose of face-to-face counselling and change of multiple lifestyle behaviours and LDL-C.

Due to the high percentage of participants who had received a face-to-face counselling session (99%), no associations with LDL-C and lifestyle behaviours could be tested.

#### Association C

The association between the dose of telephone booster calls and change of multiple lifestyle behaviours and LDL-C.

The number of telephone booster calls delivered appeared to be negatively associated with change in LDL-C and all lifestyle behaviours (see Table 4), but these associations were not statistically significant.

#### Association D

The association of the dose of the complete intervention-package as intended (at least one PRO-FIT*advice module, face-to-face counselling and at least one telephone booster call) with change in multiple lifestyle behaviours and LDL-C.

Participants who had received the complete intervention-package as intended showed improved LDL-C levels and all lifestyle behaviours, except for vegetable intake and compliance to statin therapy (see Table 4), but these associations were also not statistically significant.

## Discussion

The present paper describes the reach, dose (delivered and received) and fidelity of the PRO-FIT intervention, a combination of a web-based computer-tailored lifestyle advice (PRO-FIT*advice) and (face-to-face and telephone) counselling guided by MI. The results indicate that a representative proportion of the intended study sample agreed to participate of whom only half logged on at the PRO-FIT*advice website and completed at least one of the advice modules. Almost all participants received face-to-face counselling, however with low MI fidelity, and the majority of the planned number of telephone booster calls was delivered.

Despite its representativeness, only 34% of the people with FH invited to participate in the PRO-FIT project took part in the study. This low participation rate, as well as the StOEH screening rate, has implications for the generalizability of the results, as the sample was self-selective. Participants are likely to be more motivated to change lifestyle behaviour and our study showed significanty higher LDL-C levels in non-participants compared to participants. This is disappointing, since people with elevated LDL-C levels are most in need for a lifestyle intervention. In addition, because of the low participation rate, a decreased (cost-) effectiveness is expected on a population level [43,44]. By conducting measurements and providing counseling sessions at the participant’s home, we already tried to minimize the main burden and time investments of the participants. However, in future comparable trials, other proactive strategies to recruit high-risk participants are suggested, such as the incorporation of healthcare professionals (e.g. medical specialists or StOEH genetic field workers) during the recruitment phase, and the provision of incentives for participation.

Despite the high dose of the PRO-FIT*advice accounts delivered, the extent to which participants actively engaged in using the website as intended was disappointing. The power of web-based interventions is that they can be delivered at almost any time and anywhere, as suites the individual participant [45]. However, suboptimal exposure to web-based interventions has already been pointed out as a major concern in such health promotion studies [14]. Apparently, dose received is a less controllable process element as compared to dose delivered, which is under the control of the implementers. Robroek et al also evaluated the use of an internet-delivered behaviour change program for construction workers and found 43% of them visiting the website [46]. PRO-FIT*advice was based on the Dutch GezondLevenCheck, a quite comparable web-based tool which contains 5 (instead of 6) advice modules and is freely available to the general public and online registration before entering the advice modules is required. Comparable to PRO-FIT*advice, multiple visits to the GezondLevenCheck were possible and recommended, but not mandatory. Brouwer et al. reported a registration rate of 29% and found 91% of the registered users actually finishing at least one module [47]. This confirms that, despite the potential of PRO-FIT*advice (or web-based interventions in general) to be delivered at a high dose, achieving an acceptable dose received remains challenging and less controllable. The length of the screening questionnaires of the advice modules could have inhibited participants from completing an advice module, particularly since they overlapped with the questionnaires for evaluative purposes. In future studies on computer-tailoring, the burden of filling in (screening) questionnaires should be brought to a minimum in order to keep participants motivated, e.g. by creating a joint questionnaire, for both evaluative and tailoring purposes. Thereby, it is known that incorporating iterative feedback and interactive website components are positively associated with exposure to web-based interventions [14]. The combination of PRO-FIT*advice and personal counselling could be more successful if counsellor support is also available at an interactive communication board/forum, whereon participants also can communicate with each other. Still, the consequences of the low dose received of PRO-FIT*advice remain to be questioned, as the complete PRO-FIT intervention also incorporated face-to-face and telephone booster calls. In other words, to what extent were the gaps with regard to (un)completed advice modules and (lack of) formulated action plans, filled in by the content of the face-to-face counselling sessions?

Regarding face-to-face counselling, the dose delivered again appeared to be high, since almost all participants were visited by their personal coach. However, none of the analysed face-to-face counselling sessions met the MITI thresholds. Other studies on MI counselling have also reported below-threshold scores [48-51]. The association between MI fidelity and efficacy could not be tested in this study, but previous studies showed that a better MI performance is associated with larger intervention effects [21,52]. It has often been reported that skills required for effective MI may take longer to develop than the 3-day MI workshop in our project [53,54]. Probably, the provided MI workshop was not sufficient and more thorough monitoring and supervision of counselling skills during the intervention should have been built in. Beyond meeting MI thresholds, the face-to-face counselling sessions were part of the complete PRO-FIT intervention, and also included the discussion of the given advice at PRO-FIT*advice, and/or the (re)making of action plans. Thus, despite being a useful supplement to PRO-FIT*advice, this could have worked at the expense of fidelity to MI. Strict separation between the intervention components was impossible and undesirable.

The significant difference between the two coaches in MI fidelity, is noteworthy. By providing a 3-day workshop and an intervention protocol to both coaches, we attempted to achieve comparable delivery of MI throughout the sessions. Nevertheless, despite all effort, differences in background, demographics and other personal characteristics (e.g. counselling style) were unavoidable, and undoubtedly must have affected counselling performance. The analysed sessions showed that the coach with a more extended and diverse counselling history performed poorer than the coach with a more limited (though lifestyle counselling-) background. Literature has also shown that it has advantages to train more inexperienced coaches, e.g. students [55]. Overall, we should keep in mind that in a real-life setting, differences in the above-mentioned inter-coach characteristics are indispensable.

The secondary aim of this paper was to investigate whether the dose of: A) PRO-FIT*advice, B) face-to-face counselling, C) telephone booster calls, and D) the complete intervention-package, was associated with change in lifestyle behaviour and LDL-C levels. The delivery of the complete intervention-package as intended led to non-significant improvements in LDL-C and lifestyle behaviours. More particular, associations between the completion of the separate advice modules of PRO-FIT*advice and change in LDL-C and related lifestyle behaviours were positive, but non-significant. Other studies also showed weak or absent dose–response relationships regarding web-based lifestyle interventions [56,57]. Further, generally negative associations were found between the number of telephone booster calls and LDL-C and lifestyle behaviours, but these associations were also not statistically significant. Even if these negative associations are valid, this does not necessarily mean that the telephone booster sessions might have inhibited behavioural improvements. It may be that with fewer sessions performed, more improvements regarding lifestyle behaviours may already have been made and no further session were necessary, given that the participants were encouraged to plan the telephone sessions themselves according to their need for additional counselling.

This process evaluation has limitations. At first, the sample in this process evaluation (n = 181) might be too small to draw firm conclusions, since sample size calculations in the PRO-FIT project were based on the power to statistically detect an intervention effect [7]. Further, associations of process indicators with demographic (e.g. age), psychosocial (e.g. motivation) and behavioural (e.g. physical activity level) correlates, that could further clarify for whom the intervention works best, were not included in this process evaluation. Also, not all recommended process elements were incorporated in this process evaluation, e.g. maintenance. In general, to produce lasting effects, interventions will need to address successful intervention components/strategies that lead to sustained behavioural change. We cannot draw conclusions on the longer-term effects of the PRO-FIT intervention and the association with intervention dose. Further, the assessment of MI fidelity was limited to 20 counselling sessions, which was sufficient for determining MI quality, but made it unable to explore its association with efficacy.

Strengths of the present process evaluation include that a thorough, theory-based approach was conducted incorporating the most important process indicators. Data were mostly collected from objective sources, such as website data/coach logs. By linking these indicators to efficacy, we meet the call for more insight in the association between the process of delivery of intervention components and efficacy, contributing to a more transparent evaluation of a public health intervention and being able to indicate facilitators and barriers in translating such an intervention into practice.

## Conclusions

In conclusion, it would be feasible to implement the PRO-FIT intervention in practice, particularly PRO-FIT*advice, since it can be relative easily implemented with a high dose delivered. However, only less than half of the intervention group received the complete intervention-package as intended. Strategies to let participants optimally engage in using PRO-FIT*advice (and web-based computer-tailored interventions in general) are needed. Implementing MI in face-to-face lifestyle counselling sessions is challenging and emphasis should be put on more extensive MI training and monitoring. In order to conduct more efficacious intervention studies in the field of health promotion, we challenge fellow researchers to perform systematic process evaluations incorporating the exploration of the key process indicators reach, dose and fidelity, as well as its association with efficacy.

## Competing interests

The authors declare that they have no competing interests.

## Authors’ contributions

KB was responsible for data analysis, interpretation and reporting, while JJ held the counseling logs during the trial and assisted in data analysis. JJ, MvP, LK, JB and WvM assisted in interpreting and reporting. All authors read, edited and approved the final version of the manuscript.

## Funding

This work was supported by the Netherlands Organisation for Health, Research and Development (ZonMw) [50-50110-96-489].

## Pre-publication history

The pre-publication history for this paper can be accessed here:

http://www.biomedcentral.com/1471-2458/12/348/prepub
